# Predicting Chemotherapy Response in Triple-Negative Breast Cancer: The Role of CYP2Cs as a Biomarker and a Drug Target (A Proof-of-Concept Study)

**DOI:** 10.3390/ph19101501

**Published:** 2026-09-22

**Authors:** Nassima Zerouali, Ayesha Ahmed, Sondus Jawad, Lana Ghanem, Hevna Dhulkifle, Sara M. Abulola, Ghalya Al-Maraghi, Tamader Al-Jumaily, Lubna Therachiyil, Hanan H. Abunada, Nizar A. Al-Shar’i, Zaid H. Maayah

**Affiliations:** 1 Department of Pharmaceutical Sciences, College of Pharmacy, QU Health, Qatar University, Doha 2713, Qatar; nz1907847@student.qu.edu.qa (N.Z.);; 2 Translational Research Institute, Hamad Medical Corporation, Doha 3050, Qatar; 3 Biomedical Research Center, QU Health, Qatar University, Doha 2713, Qatar

**Keywords:** anthracycline, epoxygenase, CYP2C, triple-negative breast cancer

## Abstract

**Background:** Triple-negative breast cancer (TNBC) has a poor prognosis and limited targeted therapies, with anthracyclines being key treatments. However, many patients show reduced responsiveness to these treatments, affecting their outcomes. Arachidonic acid metabolites, specifically epoxyeicosatrienoic acids (EETs) produced by cytochrome P450 (CYP) epoxygenase enzymes, have been implicated in TNBC progression. However, limited data exist on the role of CYP epoxygenase gene expression in the responsiveness of anthracycline-based therapy in TNBC. Thus, we aimed to test the contribution of these CYPs in anthracycline responsiveness among patients with TNBC and to explore whether inhibition of CYP epoxygenase can potentiate the anti-cancer activity of anthracyclines. **Methods:** Our study used the KM-plotter database to retrieve and extract patients’ data with TNBC. The patients were assigned to two cohorts: responders or less-responders to anthracycline based on relapse-free survival. Additionally, the effects of the CYP epoxygenase inhibitor MSPPOH in combination with doxorubicin were evaluated in the TNBC cell line. **Results:** The present study shows that TNBC patients with lower responsiveness to anthracycline-based therapy exhibited higher levels of CYP2C8, CYP2C9, and CYP2C19. We also found that these CYP2Cs could be potential biomarkers for predicting anthracycline responsiveness. Using human TNBC cells, we show that a selective CYP2C epoxygenase inhibitor, MSPPOH, significantly potentiates the anti-cancer effect of anthracycline. **Conclusions:** Collectively, this study provides preliminary evidence that CYP2C epoxygenase is involved in the therapeutic response to anthracyclines in TNBC, suggesting that these enzymes may serve as predictive biomarkers. However, given the limitations of using a public database and a single TNBC cell line, continued validation within larger, independent patient cohorts is needed.

## 1. Introduction

Triple-negative breast cancer (TNBC) poses a critical therapeutic challenge because of the aggressive nature of the tumor and the lack of clear molecular targets like estrogen, progesterone, and HER-2 receptor [1]. Therefore, cytotoxic chemotherapy such as anthracycline-based regimens remains an integral component of TNBC treatment [2,3]. Mechanistically, anthracyclines like doxorubicin (DOX) kill cancer cells by intercalating with the DNA and disrupting DNA repair and replication via interfering with topoisomerase-2A, resulting in breaking the DNA and initiating caspase- and free-radical-dependent apoptosis [3,4]. Despite the effectiveness of anthracycline chemotherapy in the treatment of TNBC, a significant number of patients exhibit reduced responsiveness to anthracycline treatment, showing a notable challenge in achieving effective clinical outcomes and leading to poor prognosis [5,6]. Tailoring anthracycline therapy to the molecular profile of TNBC is necessary to improve patient outcomes and minimize the burden of overall toxicity [7,8]. This can be achieved by identifying predictive biomarkers that can stratify potential responders and spare others from ineffective therapy and unnecessary adverse effects [7,9,10]. Thus, identifying reliable indicators that can predict how TNBC patients will respond to anthracycline treatment remains an essential unmet need.

Recent integrated omics analysis unveils the important role of CYP epoxygenase as a diagnostic as well as a therapeutic target for TNBC [11]. The CYP epoxygenase enzymatic pathway encoded by CYP2B, CYP2C, and CYP2J subfamily groups generates epoxyeicosatrienoic acids (EET) [12]. EETs are known for their diverse cellular processes, including cell proliferation, migration, angiogenesis, and anti-apoptotic responses [10,12,13]. While CYP epoxygenase plays a critical role in the survival of breast cancer cells [11], there is limited literature investigating its contribution to anthracycline responsiveness in human cells and TNBC patients, as well as its potential utility as a predictive biomarker. Therefore, our study aimed to assess the role of CYP enzymes in the responsiveness of anthracyclines among patients with TNBC. We also investigated the correlation between CYP epoxygenase and key apoptotic pathway markers. Finally, we explored whether inhibiting CYP epoxygenase could potentiate the anti-cancer activity of anthracyclines.

## 2. Results

### 2.1. CYP2C Isoforms Show Moderate Potential as Predictive Biomarkers in TNBC Patients with Poor Clinical Outcomes

To investigate the clinical relevance of CYP2C epoxygenase expression in patients with TNBC, we utilized the KM-Plotter database. We first analyzed a cohort of 136 patients who had received anthracycline-based chemotherapy. The patient cohort was divided into two cohorts based on their relapse-free survival: ‘R’s (*n* = 73), who survived for over five years, and ‘LRs’ (*n* = 63), who relapsed within five years. The mean age at the initiation of anthracycline therapy for LRs was 50.2 ± 12.6 years, and for Rs, 50.6 ± 10.2 years. Further analysis showed that LRs exhibit 1.88 times higher node positivity compared to Rs. Moreover, both R and LR cohorts predominantly included patients with TNBC, with tumors graded as grade 3. Overall, this information suggests that LRs with TNBC are associated with poor clinical outcomes. Detailed patient characteristics extracted from KM-Plotter are presented in Table 1.

To address our hypothesis that CYP epoxygenases are involved in anthracycline responsiveness in patients with TNBC, we tested whether CYP genes can be used as predictive biomarkers that effectively discriminate between Rs and LRs using the ROC curve (Table 2). Within the TNBC patients, we found that the significant AUC values for CYP epoxygenase gene expressions of CYP2U1, CYP2C8, CYP2C9, and CYP2C19 are 0.6363 (*p* < 0.05), 0.6484 (*p* < 0.05), 0.6411 (*p* < 0.05), and 0.6401 (*p* < 0.05) (Table 2). We also found that the significant AUC values for the proapoptotic gene CASPASE3 are 0.661 (*p* < 0.05) and for the anti-apoptotic gene BCL2, 0.591 (*p* < 0.05) (Table 2). Given that the AUC values were relatively moderate (~0.65), we suggest that CYP2U1, 2C8, 2C9, and 2C19, along with CASPASE3 and BCL2, have the potential but limited predictive power to discriminate between LR and R groups (Table 2).

To assess the independent predictive value of CYP2C8, CYP2C9, and CYP2C19, we conducted a multivariate logistic regression analysis. The model included patient age, nodal status, and tumor grades as covariates, while the expression levels of CYP2C genes were treated as continuous variables (Table 3). Our study showed that CYP2C8, CYP2C9, and CYP2C19 remained significant even after adjusting for confounding factors, including patient age, nodal status, and tumor grade (Table 3). We have also generated ROC curves to evaluate the overall predictive performance of these multivariate models. Notably, CYP2C8, CYP2C9, and CYP2C19 showed significant AUC values of 0.78 (*p* < 0.0001), 0.77 (*p* < 0.0001), and 0.81 (*p* < 0.0001), respectively (Figure 1). Overall, our findings suggest that CYP2C8, CYP2C9, and CYP2C19 have significant independent predictive value.

### 2.2. CYP2C8, CYP2C9, and CYP2C19 Are Highly Expressed in TNBC Patients with Poor Clinical Outcomes

To assess CYP epoxygenase expression levels among TNBC patients, we compared the mean expression levels of the relevant genes between Rs and LRs (Figure 2). CYP2C8, CYP2C9, CYP2C19, CYP2U1, CASPASE3, and BCL2 were selected as they demonstrated significant AUC values (Figure 1). Our results show that CYP2C8 (Figure 2A), CYP2C9 (Figure 2B), and CYP2C19 (Figure 2C) are significantly upregulated in LRs by approximately 1.6-, 2-, and 1.8-fold compared to Rs. However, there was no significant difference in CYP2U1 expression between LRs and Rs (Figure 2D). Notably, while there was a modest upregulation in the expression level of BCL2 in LR patients compared to Rs (Figure 2E), LR patients demonstrated a significant downregulation in the expression level of the pro-apoptotic gene CASPASE3 compared to Rs (Figure 2F). Collectively, our data indicate that LRs TNBC patients with poor clinical outcomes demonstrated elevated expression levels of CYP2Cs.

### 2.3. CYP2Cs Expression Correlates with Key Apoptotic Pathway Markers

Given that the CYP epoxygenase pathway is known to promote cancer cell proliferation via inhibiting apoptosis [14], we sought to investigate the correlation between CYP 2C epoxygenase and key genes in the apoptotic pathway within the same cohort of TNBC LR patients. Notably, we found that CYP2C8, CYP2C9, and CYP2C19 demonstrated significant positive correlations with BCL2, with r values of 0.53, 0.48, and 0.52, respectively (Figure 3A–C). On the other hand, CYP2Cs showed a significant negative correlation with CASPASE3 in TNBC patients treated with anthracyclines, with r values of −0.33, −0.47, and −0.48 for CYP2C8, CYP2C9, and CYP2C19, respectively (Figure 3D–F). Overall, our observations indicate that these CYP genes may have contributed, at least in part, to the reduced responsiveness of TNBC patients to anthracycline-based therapy by modulating the apoptosis pathway.

### 2.4. Doxorubicin Treatment Upregulates the Expression of CYP2C Epoxygenase in TNBC Cells

The high expression level of CYP2C epoxygenase plays a crucial role in the proliferation and invasiveness of TNBC [15]. In this study, we examined the constitutive levels of CYP2C8, CYP2C9, and CYP2C19 in the TNBC cell line MDA-MB-231. We found that all three enzymes, CYP2C8, CYP2C9, and CYP2C19, are constitutively expressed in MDA-MB-231 cells, with CYP2C8 showing a relatively higher expression level (Figure 4A). Notably, treatment of MDA-MB-231 cells with 0.25, 0.5, and 1 µM of DOX resulted in a significant concentration-dependent upregulation of CYP2C8, CYP2C9, and CYP2C19 compared to the control (Figure 4B–D). Since 1 µM of DOX showed the highest CYP2Cs expression level, this concentration was used in all subsequent experiments.

### 2.5. Inhibition of CYP2C Epoxygenase Using MSPPOH Potentiates Doxorubicin-Mediated Cytotoxicity in TNBC Cells

Since (i) CYP epoxygenases, particularly CYP2C, have been upregulated in TNBC patients who demonstrated less responsiveness toward anthracycline-based therapy, and (ii) the expression of these CYPs was positively correlated with BCL2 and negatively correlated with CASPASE-3 in these TNBC patients, we hypothesized that CYP epoxygenase, mainly CYP2C, may play a critical role in the responsiveness toward anthracyclines. Hence, if this is the case, we predict that a selective CYP2C epoxygenase inhibitor can induce apoptosis and potentiate responsiveness to anthracyclines. Thus, we conducted our experiment using a selective CYP2C epoxygenase inhibitor, N-(methylsulfonyl)-2-(2-propynyloxy)-benzenehexanamide (MSPPOH) [16,17] on the TNBC cell line, MDA-MB-231, incubated with DOX.

Our results showed that MSPPOH alone demonstrated a modest effect on MDA-MB-231 cell viability with ~30% cell toxicity using the highest concentration of 800 µM (Figure 5A). Notably, MSPPOH significantly potentiated the effect of DOX on MDA-MB-231 cell viability in a concentration-dependent manner (Figure 5B). While 1 µM of DOX reduced 25% of MDA-MB-231 cell viability compared to control, the combination of 1 µM of DOX with 200, 400, and 800 µM of MSPPOH further reduced MDA-MB-231 cell viability by approximately 50%, 65%, and 80%, respectively, compared to control (Figure 5B). The synergistic interaction between DOX (1 µM) and MSPPOH (200, 400, and 800 µM) was assessed using the Bliss Independence model (Figure 5C,D). The combination of DOX and 200 µM of MSPPOH exhibited observed and predicted inhibitions of 50.7% and 50.5%, respectively (Bliss Δ = 0.032 ± 0.012; 95% CI [0.002, 0.063]; *p* = 0.041), suggesting an additive interaction (Figure 5C,D). Notably, a synergistic interaction was observed when DOX was combined with MSPPOH at 400 and 800 µM. The observed inhibition at 400 and 800 µM (60.9% and 81.4%, respectively) significantly exceeded the predicted inhibition (52.9% and 55.8%, respectively). This resulted in Bliss synergy scores of 0.100 ± 0.008 (95% CI [0.076, 0.123]; *p* = 0.0003) and 0.250 ± 0.001 (95% CI [0.248, 0.252]; *p* < 0.0001), indicating synergism and strong synergism at 400 and 800 µM, respectively (Figure 5C,D). Overall, these findings demonstrate that MSPPOH potentiates the cytotoxic activity of DOX in a concentration-dependent manner.

### 2.6. Inhibition of CYP2C Epoxygenase Potentiates the Anti-Cancer Effect of Doxorubicin by Inducing Apoptosis

To further understand the molecular mechanism by which MSPPOH potentiates DOX-mediated cytotoxicity in MDA-MB-231 cells, we incubated the cells with 1 µM of DOX with or without 200 µM of MSPPOH for 24 h. Thereafter, we measured the mRNA levels of apoptotic markers, including CASP3 and BAX, and anti-apoptotic markers, BCL2 and BCLxl. Our results showed that MSPPOH alone did not significantly affect the expression level of apoptotic and anti-apoptotic markers (Figure 6A–E). However, we found that 1 µM of DOX alone modestly but significantly upregulated CASP3, BCL2, BAX, and BAX/BCLxl by about 2-, 1.4-, 1.5-, and 3-fold, respectively, compared to the control. Notably, MSPPOH significantly elevated the expression of the apoptotic marker CASP3 (Figure 6A) by about 5-fold in cancer cells incubated with 1 µM of DOX. While MSPPOH did not significantly further increase BAX expression in cancer cells incubated with DOX, it significantly upregulated BAX when normalized to BCLxl compared to the DOX group (Figure 6E). On the other hand, MSPPOH significantly reduced the expression of the anti-apoptotic markers BCL2 (Figure 6B) and BCLxL (Figure 6C) in DOX-incubated cancer cells. Overall, our data show that MSPPOH potentiates DOX-mediated cytotoxicity in MDA-MB-231 cells by upregulating apoptotic markers and downregulating the anti-apoptotic markers.

To confirm the involvement of apoptosis in the anticancer effect of MSPPOH in cancer cells treated with DOX, we incubated the cells with 1 µM of DOX with or without 200 µM of MSPPOH for 24 h. Thereafter, we assessed apoptosis and cell-cycle distribution using Annexin V/propidium iodide (PI) staining and flow cytometry. In agreement with our mRNA findings, our flow cytometry data showed that MSPPOH increased the number of apoptotic cells in MDA-MB-231 cells incubated with DOX (Figure 6F). Furthermore, our cell-cycle analysis revealed that when MSPPOH was combined with DOX, there was a notable shift in the cell-cycle distribution, leading to a substantial accumulation of cells in the S phase and in the G2/M population compared to cells treated with DOX alone (Figure 6G). Overall, these results suggest that MSPPOH potentiates the effect of DOX on MDA-MB-231 cells by disrupting cell cycle progression and by inducing the apoptosis pathway.

### 2.7. Molecular Docking Evaluation of the Interaction of MS-PPOH with CYP2C8, CYP2C9, and CYP2C19

In an attempt to explain the observed biological results following treatment with MSPPOH in potentiating the anticancer effect of DOX, molecular docking was employed to investigate the binding affinity and orientation of the MSPPOH within the active site of a set of CYP450 isoforms, namely 2C8, 2C9, and 2C19. Those isoforms were selected because they were investigated in this study and have available experimentally solved X-ray crystal structures.

The argument behind this computational study is that the acetylene moiety of the MSPPOH would undergo oxidation by some of the CYP isoforms, leading to the formation of a highly electrophilic reactive ketene intermediate (R_2_C=C=O), due to the cumulated double bonds [18]. Then, an alcohol (OH) or a thiol (SH) of nearby serine, threonine, or cysteine residues would react, in situ, with the ketene by attacking the electrophilic carbonyl carbon to generate a tetrahedral intermediate that would directly and spontaneously collapse to yield an ester (RCOOR’) or a thioester (RCOSR’), ultimately leading to irreversible inhibition of the CYP enzyme [18,19], as detailed in Figure 7. This, in turn, would inhibit the CYP enzyme and compromise the production of EET, leading to re-sensitization of cancer cells to the chemotherapeutic agent.

To this end, the MSPPOH compound was modified into its ketene form and was docked into the active site of the prepared crystal structures of the three CYP450 isoforms. The binding orientation of the best-scoring pose in each of the docking experiments was investigated. The primary goal was to evaluate the possibility of forming an ester or thioester bond with the protein’s residues by measuring the distance between the carbonyl of the ketene (electrophile) and the OH or SH groups (nucleophiles) of any nearby serine, threonine, or cysteine residues. Figure 8 below shows the binding orientation (docking pose) of the MSPPOH ketene form within the CYPs’ active sites. Moreover, the docking algorithm was validated by re-docking the co-crystallized ligands into their respective binding sites, and it was successful in reproducing their native conformations with root mean square deviations (RMSDs) of the docked poses relative to their native conformations of less than 2 Å, which is within the accepted limit. Specifically, the RMSD values were 1.34 Å for the CYP2C8 crystal, 0.50 Å for the CYP2C9 crystal, and 0.75 Å for the CYP2C19 crystal.

As shown in Figure 8, the distance between the ketene carbonyl and the OH of SER100 of the CYP2C8 is 3.54 Å. This suggests a high possibility of interacting and forming an ester, hence irreversibly inhibiting this CYP isoform. Similarly, the distance between the ketene carbonyl and the OH of SER209 of the CYP2C9 is 4.74 Å. This also suggests potential interaction between the two, which could result in irreversible inhibition. On the other hand, the docked pose of MSPPOH into the CYP2C19 isoform showed a clear difference from the first two, where the distance between the ketene carbonyl and the closest nucleophile (SER209) was 10.17 Å, which is indicative of no inhibitory activity against this CYP isoform. All of these findings are consistent with the results reported in the literature [20,21].

Collectively, these findings show a potential avenue for re-sensitizing TNBC to anthracycline agents by using selective, specific CYP2C8 and CYP2C9 inhibitors as therapeutic adjuvants. However, further investigations are warranted to establish the feasibility of such a therapeutic strategy.

## 3. Discussion

One of the main challenges in treating TNBC is its aggressive nature and the high rate of resistance to chemotherapy treatment, such as anthracyclines [1,5]. In our study, we aimed to investigate the role of the CYP epoxygenase associated with reduced anthracycline responsiveness and evaluate its potential as a predictive biomarker in TNBC. Through the present study, we showed four key findings: first, TNBC patients with lower response to anthracycline-based therapy exhibited higher expression levels of CYP2C8, CYP2C9, and CYP2C19. Second, these CYP2C isoforms showed a positive correlation with the anti-apoptotic protein BCL2 and a negative correlation with the pro-apoptotic marker Caspase-3. Third, inhibition of CYP2Cs using MSPPOH potentiated the cytotoxic activity of DOX within the TNBC cell line. Finally, MSPPOH mediated its effects through a selective inhibition of CYP2C8 and CYP2C9 by a mechanism-based (suicide) inhibitor. Collectively, these data provide preliminary yet compelling evidence that the CYP2C epoxygenase pathway may contribute, at least in part, to the reduced anthracycline responsiveness phenotype in TNBC.

In the present study, we show a link between high CYP2C expression and the regulation of key apoptotic proteins in TNBC patients with a lower response to anthracyclines, suggesting that the underlying mechanism includes a cell-survival-dependent resistance pathway. CYP epoxygenases, such as CYP2C enzymes, metabolize arachidonic acid into bioactive epoxy fatty acids called EETs [2]. EETs are well known to induce angiogenesis, inhibit apoptosis, promote tumor growth, stimulate cell proliferation, and cause cancer cell metastasis [12,13]. In our study, we found that CYP2C expression in TNBC patients with lower response to anthracycline therapy was positively correlated with the anti-apoptotic gene BCL2 and negatively correlated with the pro-apoptotic gene Caspase-3, suggesting a possible mechanistic link. Given that EETs were shown to promote cell survival by upregulating anti-apoptotic genes while downregulating pro-apoptotic genes [22,23], we assume that CYP2Cs may contribute, at least in part, to the lower response to anthracycline therapy via increasing the level of arachidonic acid epoxygenase metabolites, EETs [14]. Supporting this notion, it has been shown that higher expression levels of CYP epoxygenases, such as CYP2C, and elevated EET levels were associated with the aggressiveness and invasive phenotype of breast cancer [15]. Moreover, overexpression of CYP epoxygenase promoted the metastasis of MDA-MB-231 human breast cancer cells to the lungs of BALB/C athymic mice [24]. Conversely, silencing CYP epoxygenases reduces TNBC cell proliferation [15]. Furthermore, lower tumorigenesis and reduced EET levels were observed in athymic nude mice injected with A549 cells expressing a loss-of-function phenotype of CYP2C9, CYP2C9*2, and CYP2C9*3, compared to the mice injected with the A549 cells expressing empty adenovirus [25]. Thus, we hypothesized that selective targeting of CYP2C epoxygenase may induce apoptosis and improve responsiveness to anthracycline treatment in TNBC.

In the present study, we found that a selective inhibitor of CYP2C epoxygenase, MSPPOH [16,17], significantly increased the sensitivity of the TNBC cell line MDA-MB-231 to DOX. Consistent with our findings, MSPPOH has been shown to substantially decrease prostate cancer cell invasion, migration, and motility by inhibiting CYP epoxygenases such as CYP2C and reducing EETs production [17]. Of interest, the exogenous administration of EETs significantly abolished the effect of MSPPOH on cell invasion, suggesting that the anticancer effect of MSPPOH is mediated through the suppression of CYP2C/EETs-dependent signaling [17]. Similarly, silencing CYP2C or inhibiting CYP epoxygenase with 17-ODYA or 1,2-di-O-α-linolenoyl-3-O-β-galactopyranosyl-sn-glycerol (dLGG), a bioactive phytogalactolipid, reduced EETs concentration and significantly reduced the aggressiveness and metastasis of TNBC cells [11,26]. In agreement with this observation, female non-small cell lung cancer patients with loss-of-function CYP2C9 SNPs, such as CYP2C9*2 and CYP2C9*3, demonstrate better survival, reduced tumorigenesis, and lower EET levels [25]. Collectively, we suggest that high CYP2C expression may contribute to reduced DOX responsiveness in TNBC by increasing EET levels. Nevertheless, we could not rule out the possibility that CYP2Cs may contribute to chemoresistance by directly metabolizing DOX [27]. However, given that CYP2Cs, unlike CYP3A4, play a negligible role in the metabolism of DOX, it is likely that CYP2Cs may contribute to chemoresistance in TNBC via EET signaling [13].

Using molecular docking, we investigated the binding affinity and orientation of MSPPOH within the active sites of CYP2C. A previous study demonstrated that the carbon chain backbone of MSPPOH resembles the length and flexibility of the arachidonic acid structure [28]. Also, the N-(methylsulfonyl)amide group of MSPPOH mimics the carboxylic acid head of arachidonic acid [28]. Thus, MSPPOH can bind to the same catalytic site on CYP2C that arachidonic acid normally occupies [28]. Upon attaching to the binding pocket, CYP2C oxidizes MSPPOH to form a ketene intermediate that can form an ester upon interaction with nearby nucleophiles such as serine or cysteine, thereby irreversibly inhibiting the CYP2C isoform [18,19]. Given that we show that CYP2C8 and CYP2C9 isozymes are able to maintain similar spatial orientation required for this self-destruction oxidation, we suggest that MSPPOH is a selective inhibitor of CYP2C8 and CYP2C9 via a mechanism-based (suicide) inhibition [16]. Overall, our observations imply a potential avenue for re-sensitizing TNBC to anthracycline agents by using selective, specific CYP2C8 and CYP2C9 inhibitors as therapeutic adjuvants. However, further investigations are warranted to establish the feasibility of such a therapeutic strategy.

Our findings are consistent with increasing evidence implicating the CYP epoxygenase pathway in the progression and the aggressiveness of TNBC. For instance, previous findings by Apaya et al. (2018) unveil the CYP epoxygenase pathway as a promising therapeutic target in TNBC [11]. In the present study, we demonstrated that CYP epoxygenases, particularly CYP2Cs, may serve as predictive biomarkers and drug targets for reduced anthracycline responsiveness in TNBC patients. However, the predictive performance, as assessed by ROC analysis, was only modest. Thus, although the AUC values were statistically significant, the biomarker status remains preliminary, and the biomarker potential of these genes should be interpreted with caution.

An important limitation of the present study is that clinical analysis was based on a retrospective analysis of a public database with a relatively small sample size [9]. Given that patient age, nodal status, and tumor grade were available in the database, the logistic regression models were adjusted only for these covariates. We explicitly acknowledge that other potentially relevant clinical confounders available in real-world cohorts (e.g., treatment regimens, anthracycline dose, stage, and tumor molecular heterogeneity) could not be evaluated [9]. Thus, future studies should comprehensively consider all these covariates. Since the predictive performance reported by ROC analysis was only modest, the biomarker value remains preliminary. Another limitation of our study is that the experimental testing was also limited to a single TNBC cell line, which reduces generalizability. Moreover, given that CYP2C/EETs play an integral role in protecting the cardiovascular system [29], it is plausible to consider delivering a selective CYP2C inhibitor directly to cancer cells rather than administering it systemically in order to reduce potential cardiotoxicity. Finally, we did not measure EET levels directly either in patient tumors or in our cell culture model.

## 4. Methods

### 4.1. The Kaplan–Meier Plotter Database Analysis

The Kaplan–Meier (KM) Plotter Database is an online platform that is a valuable tool for identifying predictive biomarkers in breast cancer (https://kmplot.com/analysis/) (Accessed on 1 November 2023) [9]. This database utilizes multiple transcriptome-level gene expression data of 3104 breast cancer patients with treatment and response data. Additionally, it is one of the first databases that serves as a genome-wide validation tool for potential future predictive biomarkers [9]. Hence, our study used the KM-Plotter database to retrieve and extract patient data. We specifically queried the database for patients with TNBC treated with anthracyclines. The characteristics and diversity of the studies incorporated into the KM-Plotter database are summarized in Table 4. Notably, the studies span from 2005 to 2015 and originate from various locations, including the USA, UK, Spain, Slovenia, Russia, and numerous other countries (Table 4) [9]. The countries and the time frame of the studies were not selected by the authors but were obtained from the KM-Plotter dataset [9].

The main data obtained from the KM-Plotter database include relapse-free survival classification, gene expression levels, nodal status, tumor grade, and patient age. The nodal status indicates whether lymph node metastasis is present (positive) or absent (negative). We categorized the unavailable nodal status data as unknown. The tumor grade was obtained from the KM-plotter database and categorized as 1, 2, or 3 based on tumor aggressiveness. The patients were stratified into two cohorts based on relapse-free survival: those with a relapse-free survival over 5 years were considered Rs (*n* = 73), and those who relapsed within 5 years were classified as less-responders (LR) (*n* = 63). The sample size (*n* = 136 total, Rs = 73, LRs = 63) was determined based on the number of eligible TNBC patients available in the KM-Plotter (Table 4) [9].

Receiver operating characteristic (ROC) curve analysis was performed to test the ability of selected CYP epoxygenase and apoptosis-related gene expression levels to discriminate between Responders and Less-Responders. The Affymetrix IDs used for CYP genes are listed in Table 4. Our inclusion criteria involved any patients with TNBC who received any type of anthracycline therapy, and their relapse-free survival and CYP epoxygenase and apoptosis-related gene expression data were available. We excluded patients who did not receive any type of anthracycline therapy and lacked information on relapse-free survival. Given that our analysis depends on data collected from KM-Plotter, we acknowledge this as a limitation of the study.

### 4.2. Cell Culture and Treatment

The human TNBC MDA-MB-231 cell line “(cat. no. HTB-26; American Type Culture Collection)” was utilized in this research and maintained as described previously [29]. The experiments were conducted in accordance with international guidelines and the policies required by Qatar University and the Department of Research in the Ministry of Public Health for the use of cell culture in experimental studies under the approval of the Institutional Biohazard Committee (approval no. QU-IBC-2023/045; Doha, Qatar).

### 4.3. Cell Viability

All treatments were conducted under humidified conditions at 37 °C with 5% CO_2_. The 3-[4,5-dimethylthiazol-2-yl]-2,5-diphenyltetrazolium bromide (MTT) assay was utilized to measure the influence of DOX and N-methylsulfonyl-6-(2-propargyloxyphenyl) hexanamide (MSPPOH) on cell viability as described previously [29]. The effects of DOX and MSPPOH were evaluated by measuring the ability of live cells to convert MTT to coloured formazan crystals. Cell viability was calculated as a percentage relative to control wells, which were labeled as 100% viability.

### 4.4. Flow Cytometry

Apoptosis was analysed using Dual Staining of Annexin V and Propidium iodide (PI) (BD Biosciences, San Jose, CA, USA) according to the procedure reported by the manufacturer, as described previously [29]. Briefly, we incubated MDA-MB-231 cells with 1 μM DOX with or without 200 μM MSPPOH for 24 h. After that, the cells were washed with phosphate-buffered saline (PBS) and reconstituted in the dark with a FITC-labelled annexin V and PI-binding solution for 15 min and measured using a flow cytometer.

### 4.5. Quantitative Real-Time PCR (qRT-PCR)

Total RNA was extracted from MDA-MB-231 cells using the TRIzol reagent (Invitrogen^®^, Carlsbad, CA, USA), following previously established protocols [43]. A NanoDrop spectrophotometer was used to measure the purity and concentration of the total RNA. The corresponding cDNA from the RNA was synthesized using the High-Capacity cDNA RT kit (Applied Biosystems; Thermo Fisher Scientific, Inc, Waltham, MA, USA) based on the manufacturer’s instructions. Quantification of mRNA expression was carried out using the QuantStudio 5 system. Luna^®^ Universal qPCR Master Mix reagent (New England Biolabs, Ipswich, MA, USA) was utilized during our experiment. The primers used for RT-qPCR were obtained from Thermo Fisher Scientific, Inc. The primer sequences used in the study are listed in Table 5. The ΔΔCt method was applied for analyzing qRT-PCR data as described previously [43]. The reference gene β-Actin was used to normalize the mRNA expression fold changes. The 2−△CT formula was utilized to calculate the constitutive level of CYP2C8, CYP2C9, and CYP2C19 in MDA-MB-231 cells.

### 4.6. Computational Study of MSPPOH

The crystal structures of the selected CYP enzymes were retrieved from the Protein Data Bank (PDB). Their accession codes are 2NNJ for CYP2C8 (resolution of 2.28 Å), 1R9O for CYP2C9 (resolution of 2.00 Å), and 4GQS for CYP2C19 (resolution of 2.87 Å). All crystal structures were prepared using the prepare protein protocol in Discovery Studio^®^ 2025 (DS; Biovia, Dassault Systèmes S.E., Vélizy-Villacoublay, France), and the binding sites were defined with spheres of radii 8–8.5 Å based on the co-crystallized ligands. The chemical structure of MSPPOH was downloaded from PubChem, then it was modified into its ketene form using ChemDraw Professional 16.0, and was prepared using the prepare ligands protocol in DS. Then, it was docked into the active sites of the prepared crystal structures using the CDOCKER docking protocol in DS. The docking protocol was validated by redocking the co-crystallized ligands into their respective binding sites and measuring the root mean square deviations (RMSD) of the generated poses compared to their native binding conformation.

### 4.7. Statistical Analysis

Statistical analyses were conducted using GraphPad Prism software (version 10.1) from GraphPad Software, Inc., La Jolla, CA, USA. The comparison between the two patient cohorts (Rs and LRs) was performed using the Mann–Whitney test and the Receiver Operating Characteristic test (ROC). Our data on patient characteristics, extracted from KM-Plotter, are reported as mean ± SD. The correlation between these CYP gene expression levels and the anti-apoptotic/pro-apoptotic gene expressions was assessed using Pearson’s Correlation coefficient. We performed a multivariate logistic regression analysis to address potential confounding factors. To determine which treatment group(s) significantly differed from the DOX group in the cell culture experiment, we performed a one-way analysis of variance (ANOVA) followed by Tukey–Kramer’s post hoc multiple-comparison test. The Bliss independence model was used to evaluate the synergistic interaction between DOX and MSPPOH. Prior to the analysis, we normalized the cell viability data to the untreated control group. The following equation was used to calculate the expected fractional effect of each combination: “Fa(expected) = Fa(DOX) + Fa(MSPPOH) − Fa(DOX) × Fa(MSPPOH), where Fa represents the fraction of cells affected” [44]. We obtained the Bliss synergy score from the difference between the expected and the observed fractional inhibition values. A bliss synergy score of Δ > +0.1 indicates a synergistic interaction, whereas a score between −0.1 and +0.1 suggests an additive interaction. The score Δ was presented as mean ± SEM. The statistical significance was determined using a one-sample *t*-test (GraphPad Prism software (version 10.1)). *p*-values less than 0.05 were considered statistically significant.

## 5. Conclusions

In conclusion, we found that TNBC patients with a lower response to anthracycline-based therapy exhibited higher CYP2C expression levels. Notably, inhibition of CYP2Cs using MSPPOH potentiated the cytotoxic activity of DOX within the TNBC cell line. Mechanistically, MSPPOH causes a selective inhibition of CYP2C8 and CYP2C9 by a mechanism-based (suicide) inhibitor. Overall, this study provides preliminary evidence that CYP2C epoxygenase is involved in the therapeutic response to anthracyclines in TNBC, suggesting that these enzymes may serve as potential predictive biomarkers. However, given the limitations of using a public database and a single TNBC cell line (MDA-MB-231), the findings remain preliminary, and further validation in larger, independent patient cohorts and additional TNBC models is needed.

## Figures and Tables

**Figure 1:**
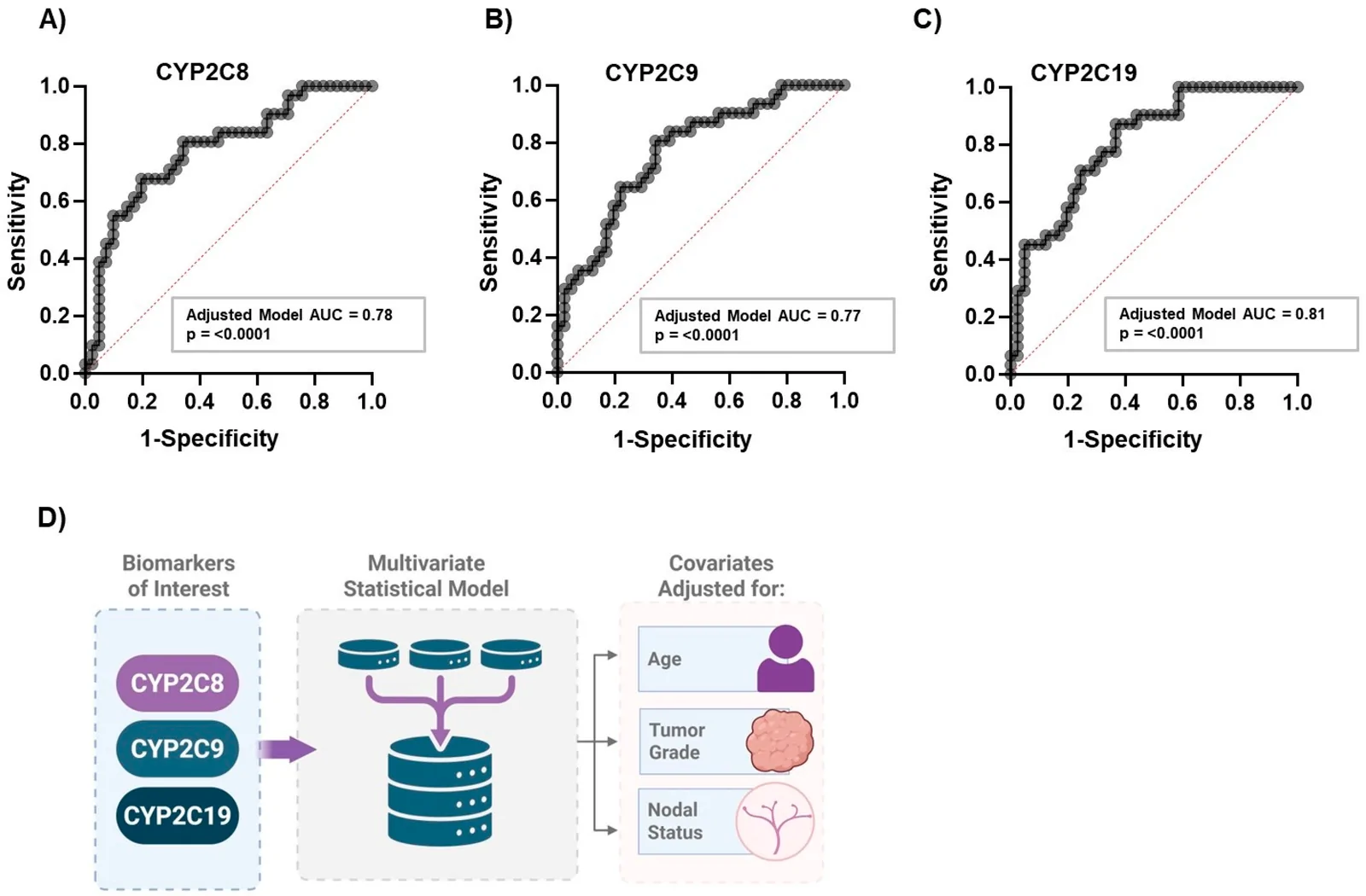
Receiver Operating Characteristic (ROC) curves for the multivariate predictive models of CYP2Cs. (**A**) CYP2C8, (**B**) CYP2C9, (**C**) CYP2C19. ROC curves demonstrate the predictive accuracy of the multivariate logistic regression models for anthracycline response. (**D**) Models include individual gene expression alongside patient age, nodal status, and tumor grade.

**Figure 2:**
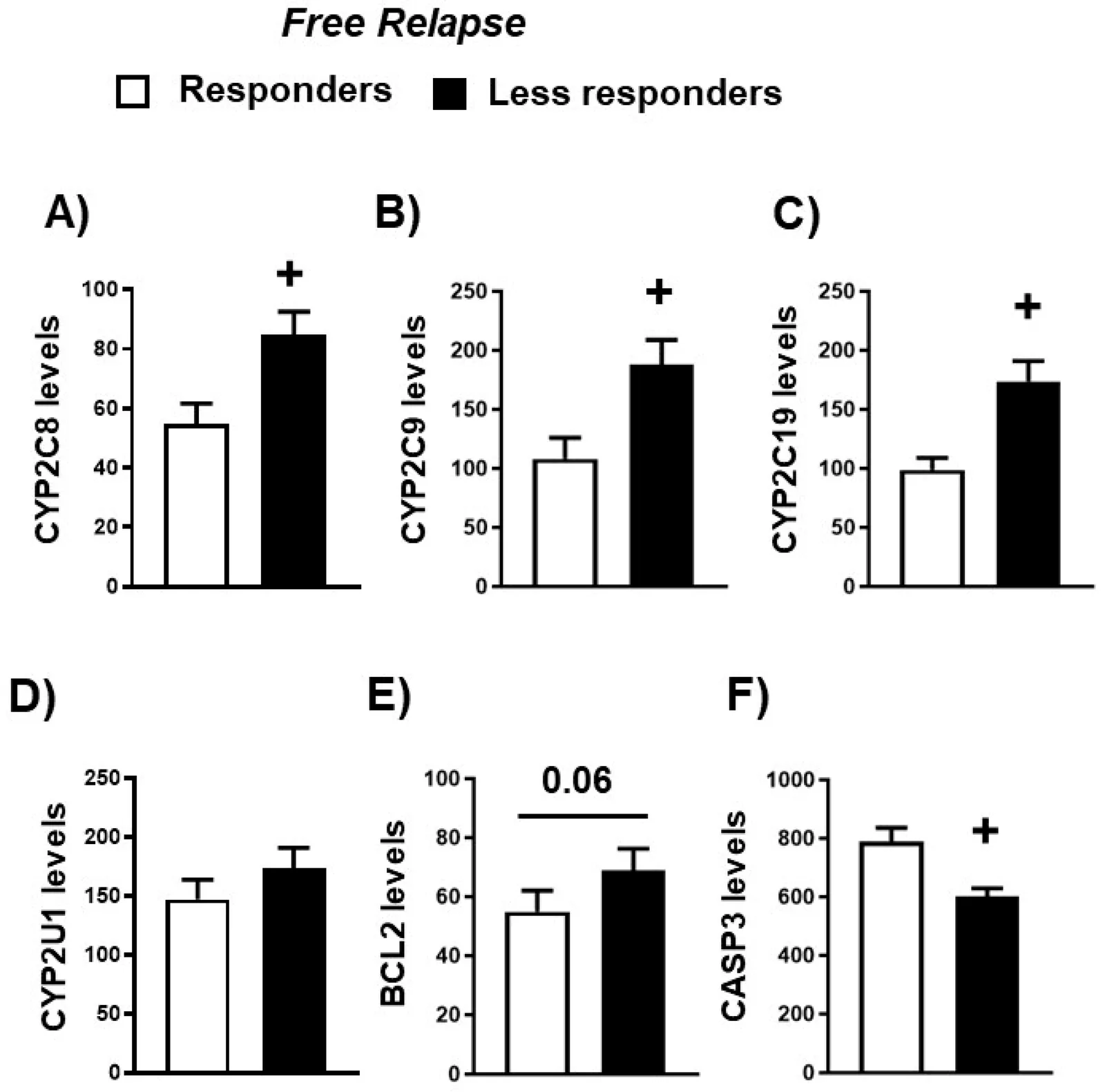
CYP2C Epoxygenase Expression in Responders and Less-Responders of Anthracycline-based Therapy in TNBC Patients. The KM-plotter database was used to retrieve and extract patients with TNBC. The mRNA expression level of CYP epoxygenase and apoptotic markers. (**A**) CYP2C8, (**B**) CYP2C9, (**C**) CYP2C19, (**D**) CYP2U1, (**E**) BCL2, and (**F**) Caspase 3 (CASP3). A Mann–Whitney test was used to compare the two groups. + *p* < 0.05 vs. control.

**Figure 3:**
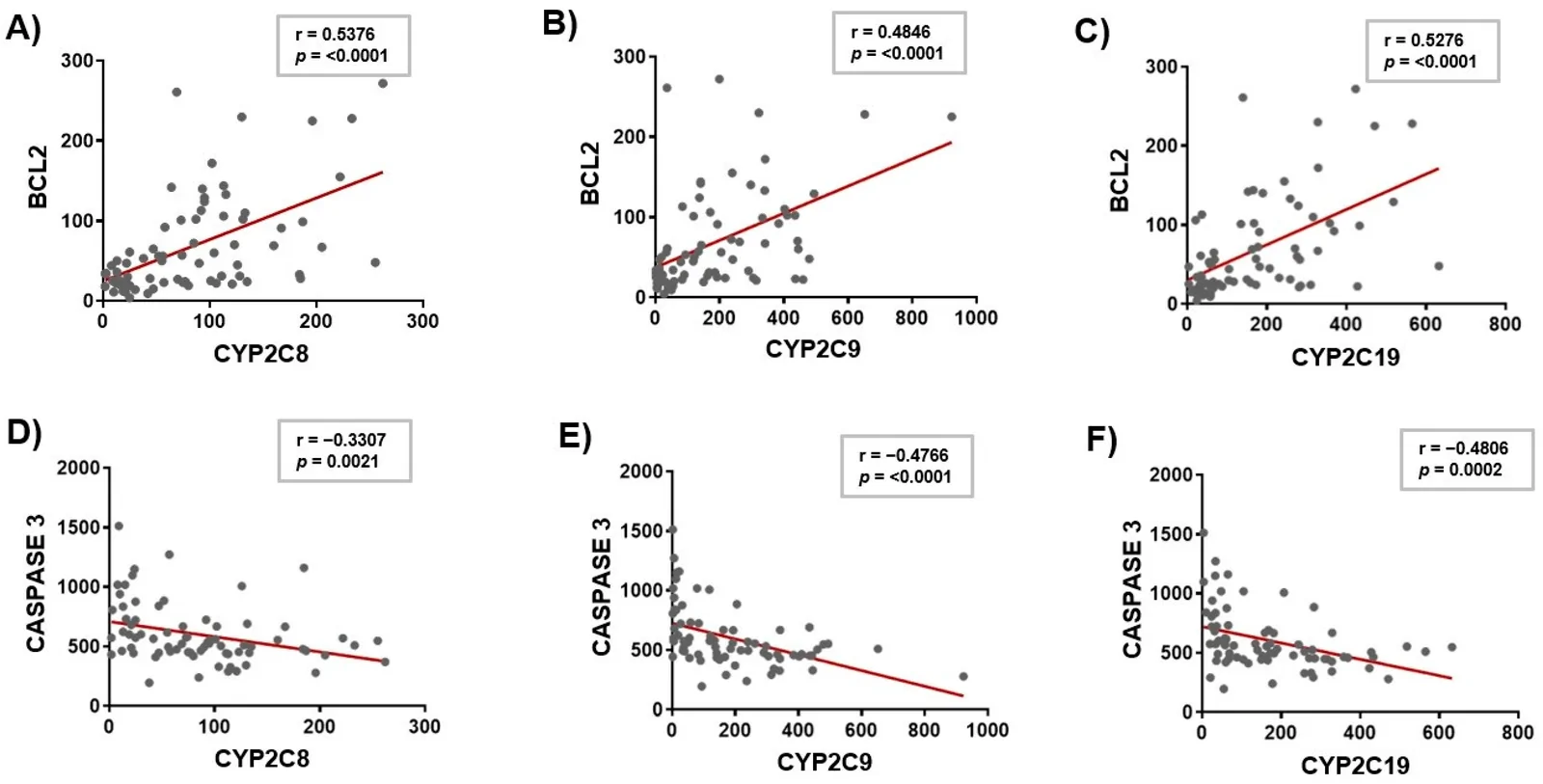
Correlation between CYP2Cs and apoptotic markers, BCL2 and Caspase 3 (CASP3). Correlation between (**A**) CYP2C8, (**B**) CYP2C9, or (**C**) CYP2C19 and BCL2. Correlation between (**D**) CYP2C8, (**E**) CYP2C9, or (**F**) CYP2C19 and CASPASE3 (CASP3). Pearson’s correlation coefficient was used to determine the correlations between CYP levels and apoptotic markers.

**Figure 4:**
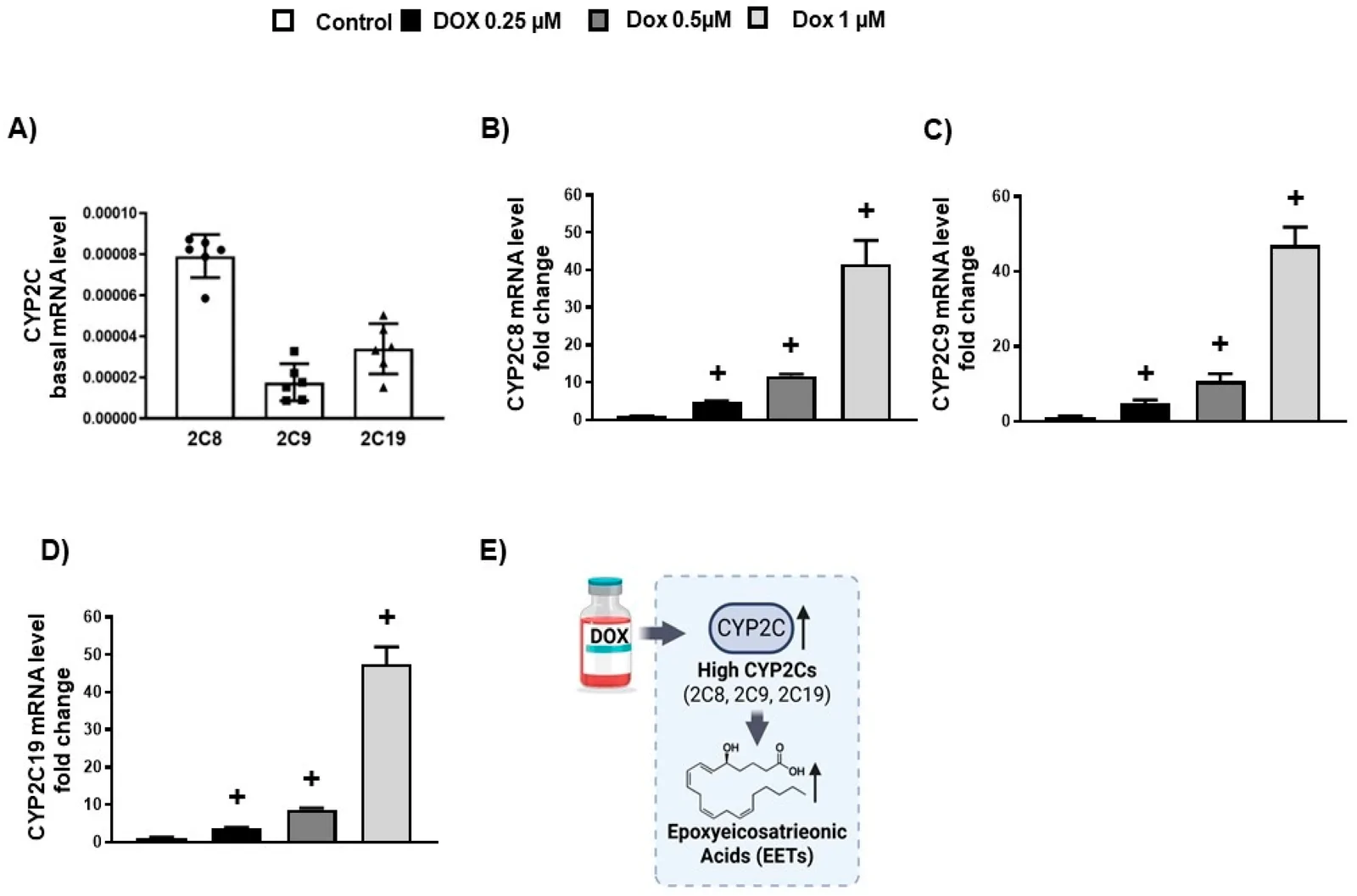
Doxorubicin upregulated the expression of CYP2C8, CYP2C9, and CYP2C19 in Triple-Negative Breast Cancer Cells. Human triple-negative breast cancer cells, MDA-MB-231 cells, were treated with 0.25, 0.5, and 1 µM of doxorubicin (DOX) for 24 h. (**A**) Constitutive level of CYP2C8, CYP2C9, and CYP2C19 mRNA in untreated MDA-MB-231 cells (individual dots represent biological replicates). (**B**–**D**) Quantification of mRNA expression levels by qRT-PCR. (**B**) CYP2C8, (**C**) CYP2C9, and (**D**) CYP2C19. (**E**) The upregulation of CYP2C enzymes by DOX will lead to an increased level of epoxyeicosatrienoic acids (EETs). Results are shown as means ± SEM. We used a one-way analysis of variance (ANOVA) followed by Tukey–Kramer’s post hoc multiple-comparison test.  + *p* < 0.05 vs. control.

**Figure 5:**
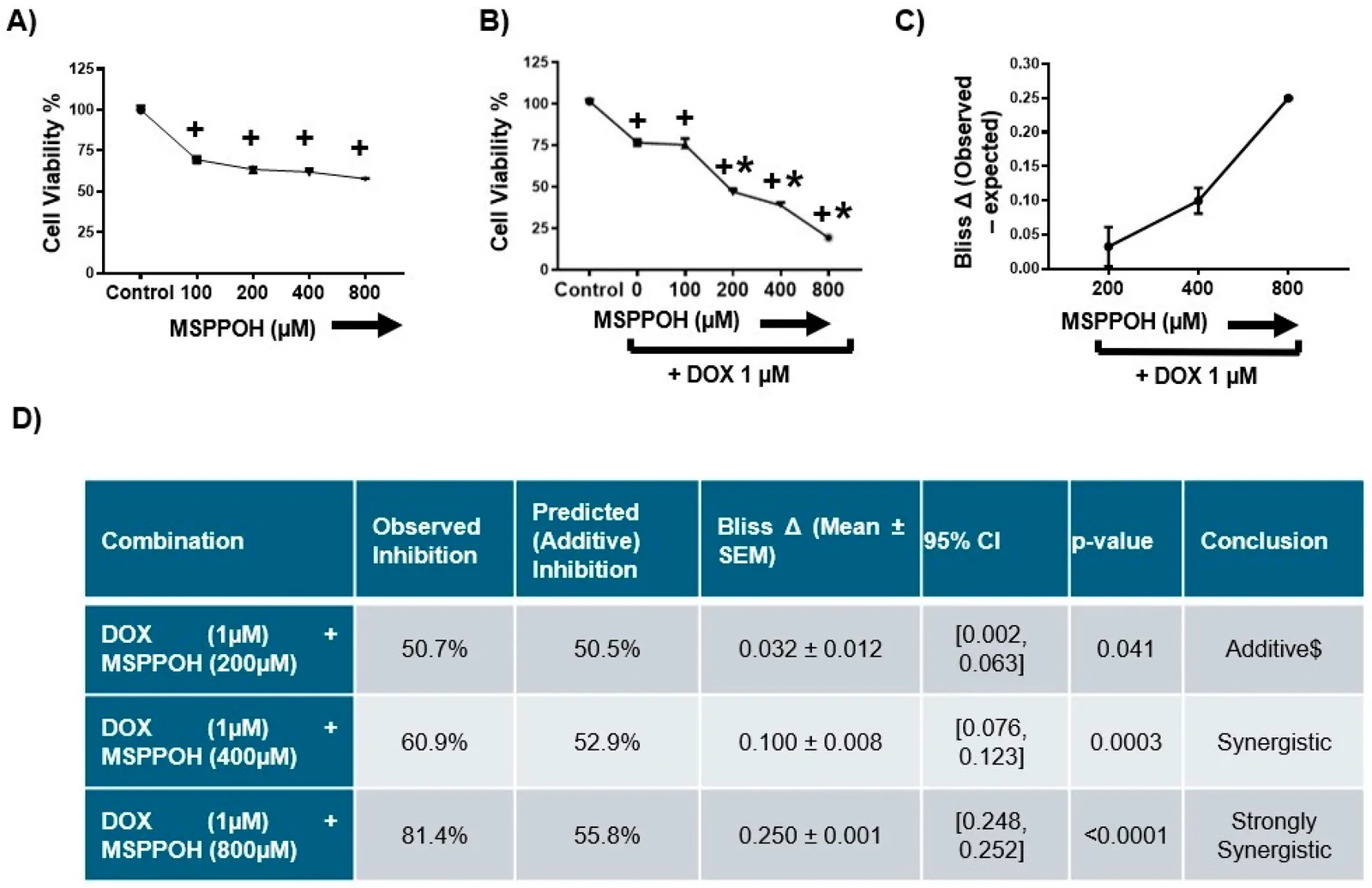
CYP2C Epoxygenase Inhibitor, MSPPOH, Potentiates DOX-Induced Cell Toxicity in Triple-Negative Breast Cancer Cells. Human triple-negative breast cancer cells, MDA-MB-231 cells, were treated with 200, 400, and 800 µM of MSPPOH and/or 1 µM of doxorubicin (DOX) for 24 h. (**A**,**B**) Cell viability using the MTT assay. Cell viability was expressed as a percentage of the untreated control. While 1 µM of DOX reduced 25% of MDA-MB-231 cell viability compared to the control, the combination of 1 µM of DOX with 200, 400, and 800 µM of MSPPOH further reduced MDA-MB-231 cell viability by approximately 50%, 65%, and 80%, respectively, compared to the control (+ *p* < 0.05 vs. control, * *p* < 0.05 vs. DOX). (**C**,**D**) Synergy Analysis of DOX and MSPPOH combinations using the Bliss Independence model: Δ > +0.1 = synergy; −0.1 to +0.1 = additive. The statistical significance was determined using a one-sample *t*-test. $ Statistically significant but Bliss Δ below synergy threshold; classified as additive.

**Figure 6:**
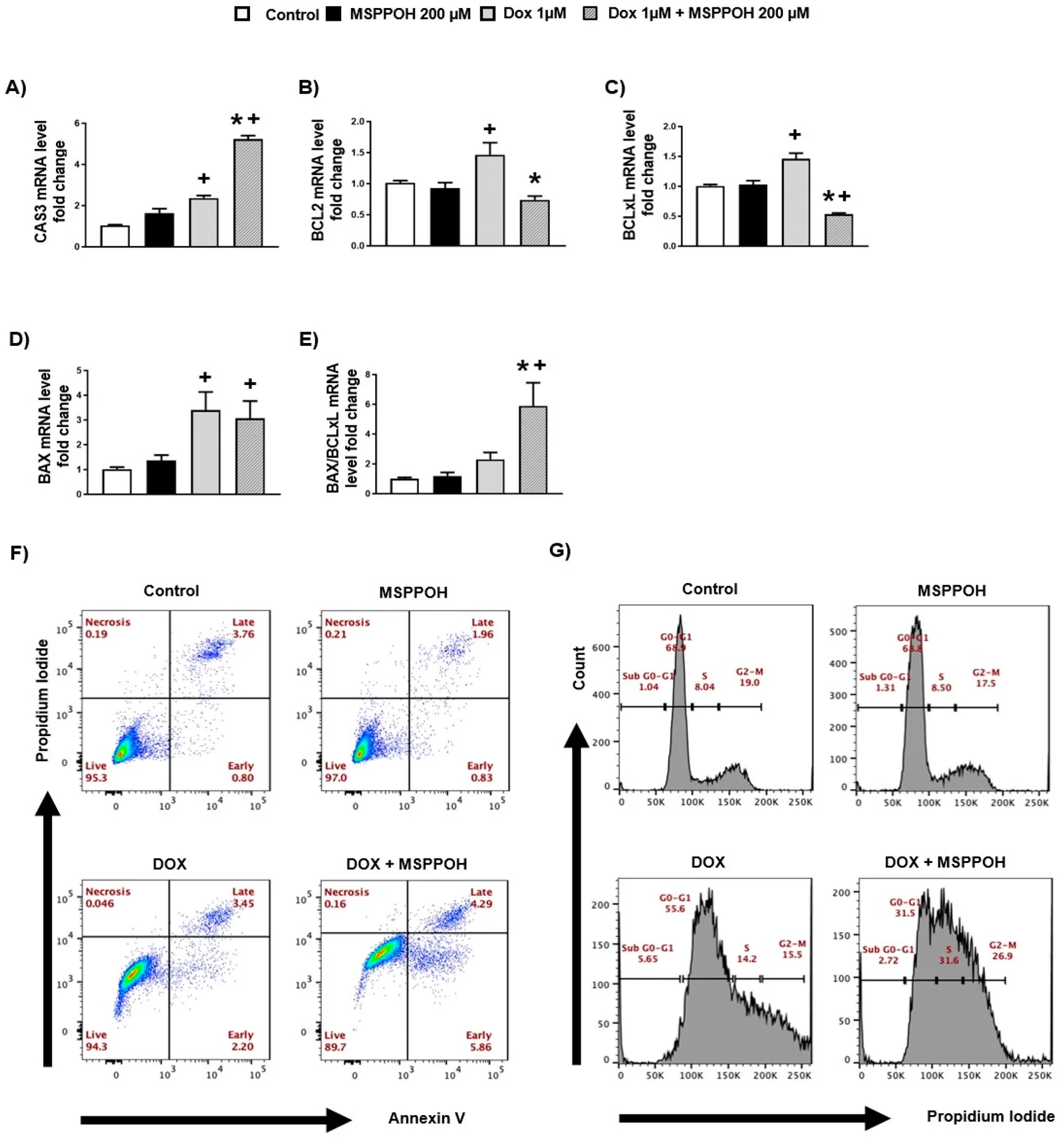
CYP2C Epoxygenase Inhibitor, MSPPOH, Potentiates DOX-Induced Apoptosis in Triple-Negative Breast Cancer Cells. (**A**–**E**) Human triple-negative breast cancer cells, MDA-MB-231 cells, were treated with 200 µM of MSPPOH and/or 1 µM of DOX for 24 h. Quantification of mRNA expression levels by qRT-PCR. (**A**) CASPASE3 (CASP3), (**B**) BCL2, (**C**) BCLxL, (**D**) BAX, and (**E**) Ratio of BAX/BCLxL. Results are shown as means ± SEM. We used a one-way analysis of variance (ANOVA) followed by Tukey–Kramer’s post hoc multiple-comparison test.  + *p*  <  0.05 vs. control; * *p*  <  0.05 vs. DOX. (**F**,**G**) Human triple-negative breast cancer cells, MDA-MB-231 cells, were treated with 200 µM of MSPPOH and/or 1 µM of DOX for 24 h. (**F**) Annexin V/propidium iodide (PI) staining and flow cytometry were used to assess apoptosis in the cells. (**G**) Cell cycle analysis using flow cytometry.

**Figure 7:**
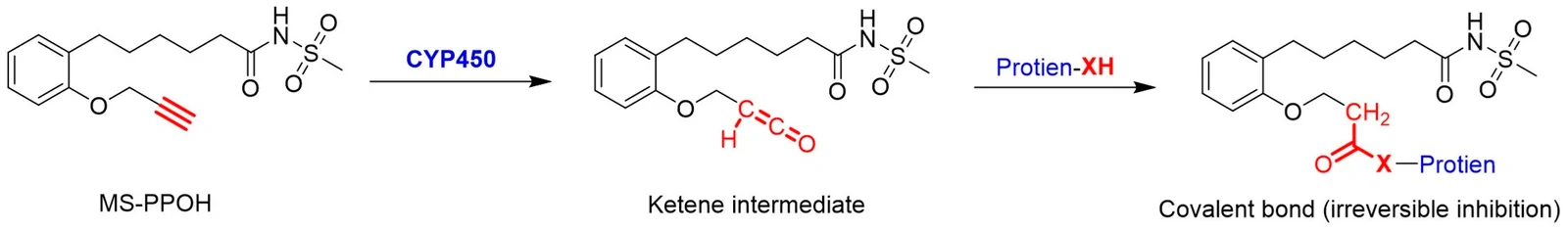
Oxidation of MS-PPOH by a CYP enzyme to form a ketene intermediate that can be trapped by reacting with protein-XH, where X=O (alcohol) or S (thiol), to form an ester or thioester.

**Figure 8:**
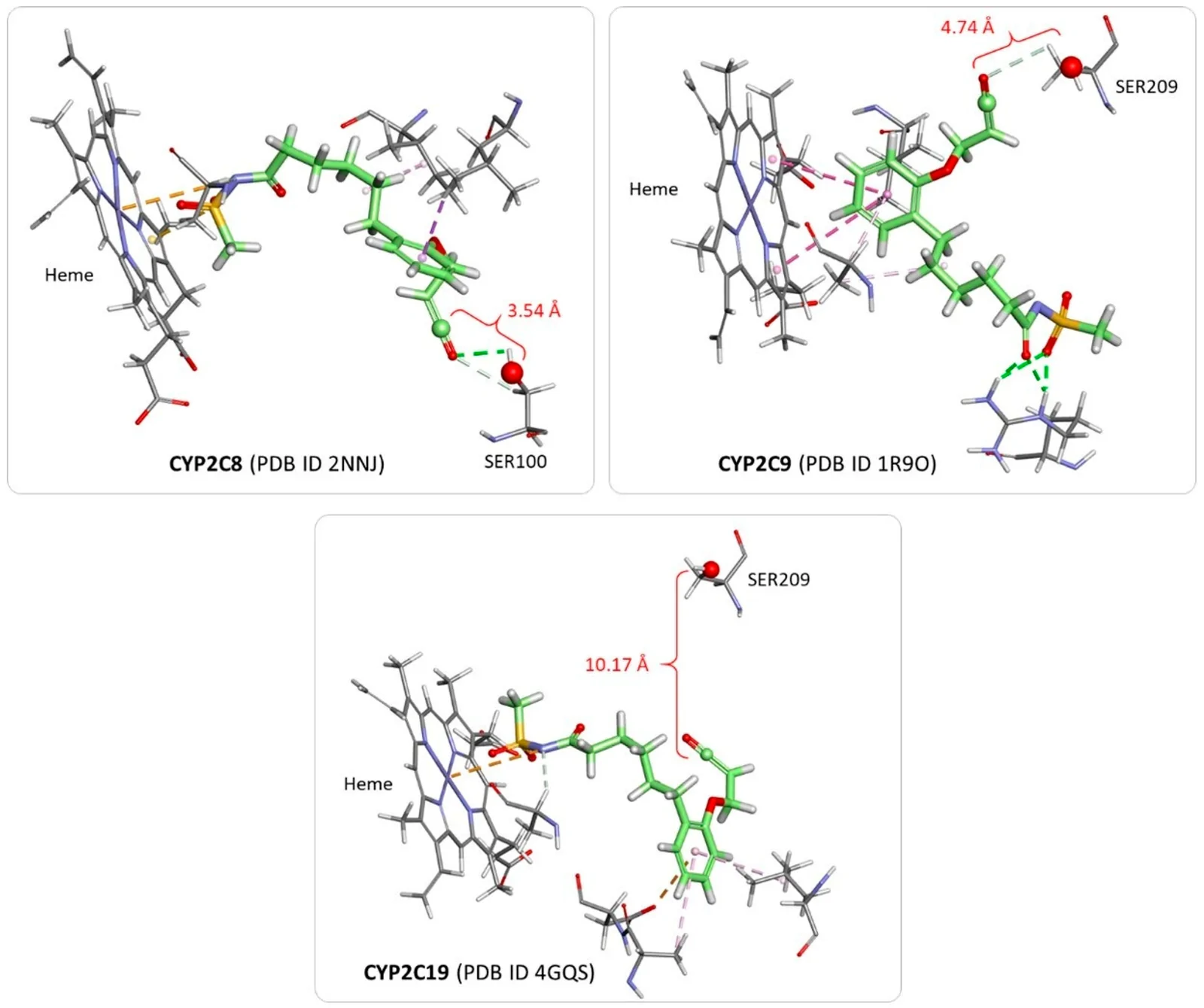
The binding orientation of MS-PPOH ketene forms within the active sites of different CYP isoforms. Enzymes’ amino acid residues are shown as sticks. MS-PPOH ketene is shown as sticks with carbons colored pale green. The hydroxyl oxygen of the serine residues and the carbonyl carbon of ketene are shown as red and pale green spheres, respectively, and the distance between them is highlighted.

**Table 1:** Characteristics of non-responders and responders TNBC patients from KM-Plotter.

Response from the Patient	Age (Mean ± SD)	Nodal Status (+/−/Unknown) (%)	Grade (1/2/3) (%)
Less-Responder (*n* = 63)	50.2 ± 12.6	65.75/30.14/4.11	0.00/20.00/80.00
Responder (*n* = 73)	50.6 ± 10.2	34.92/47.62/17.46	0.00/10.00/90.00

**Table 2:** AUC values from the ROC curves of CYP Epoxygenase Expression of Less-responders and Responders of Anthracycline-based therapy in TNBC patients.

Type	Symbol	Affymetrix ID	Name	Number of Less-Responders	Number of Responders	AUC (*p*-Value)
Epoxygenase	CYP2U1	226402_at*	Cytochrome P450 family 2 subfamily U member 1	24	37	0.6363 (0.0280)
	CYP2B6	206755_at	Cytochrome P450 family 2 subfamily B member 6	73	63	0.5633 (0.1000)
	CYP2C8	208147_s_at	Cytochrome P450 family 2 subfamily C member 8	73	63	0.6484 (0.0009)
	CYP2C9	220017_x_at	Cytochrome P450 family 2 subfamily C member 9	73	63	0.6411 (0.0015)
	CYP2C19	216058_s_at	Cytochrome P450 family 2 subfamily C member 19	73	63	0.6401 (0.0015)
	CYP2E1	209975_at	Cytochrome P450 family 2 subfamily E member 1	73	63	0.5472 (0.1700)
	CYP2J2	205073_at	Cytochrome P450 family 2 subfamily J member 2	73	63	0.5381 (0.2200)
	CYP2S1	223385_at*	Cytochrome P450 family 2 subfamily S member 1	73	63	0.5726 (0.1700)
Apoptotic Genes	BCL2	207005_s_at	B-cell lymphoma 2	73	63	0.591 (0.0330)
	BCL2L1 (BCLxL)	215037_s_at	B-cell lymphoma 2 like 1	73	63	0.568 (0.0860)
	BAX	211833_s_at	BCL2-Associated X protein	73	63	0.556 (0.1300)
	CASP3	202763_at	Cysteinyl aspartate proteases	73	63	0.661 (0.0004)

**Table 3:** Multivariate Logistic Regression Analysis for Predicting Treatment Response.

Variable	Odds Ratio	95% Confidence Interval	*p*-Value
**Model 1: CYP2C8**			
CYP2C8 Expression	0.988	0.978–0.997	0.0135
Age	1.020	0.973–1.071	0.4093
Nodal status (Positive vs. Negative)	0.363	0.108–1.134	0.0870
Grade (Grade 3 vs. Grade 2)	7.613	1.069–164.6	0.0862
**Model 2: CYP2C9**			
CYP2C9 Expression	0.996	0.992–0.999	0.0368
Age	1.022	0.976–1.072	0.3498
Nodal status (Positive vs. Negative)	0.367	0.113–1.119	0.0832
Grade (Grade 3 vs. Grade 2)	7.265	1.030–154.2	0.0910
**Model 3: CYP2C19**			
CYP2C19 Expression	0.990	0.983–0.995	0.0020
Age	1.026	0.978–1.078	0.2967
Nodal status (Positive vs. Negative)	0.365	0.101–1.215	0.1074
Grade (Grade 3 vs. Grade 2)	8.492	1.013–205.3	0.0902

**Table 4:** Characteristics of the Studies involved in TNBC in the KM-Plotter Database in Free-Relapse.

References	Year	Country
[30]	2005	Sweden
[31]	2008	Belgium
[32]	2010	Switzerland
[33]	2010	USA
[34]	2010	USA
[35]	2010	USA
[36]	2011	USA
[37]	2011	Germany
[38]	2011	EBCC and USA
[39]	2011	-
[40]	2012	-
[41]	2013	Sweden, Netherlands, Slovenia, Italy, Germany, USA, France, UK, Belgium, Singapore
[42]	2015	France

**Table 5:** Primer sequences for qRT-PCR.

Primer Name	Forward Sequence	Reverse Sequence
h-β-ACTIN	CTGGCACCCAGCACAATG	GCCGATCCACACGGAGTACT
h-CASPASE-3	GAGTGCTCGCAGCTCATACCT	CCTCACGGCCTGGGATTT
h-BAX	CCCTTTTGCTTCAGGGTTTC	TCTTCTTCCAGATGGTGAGTG
h-BCL-XL	GGCGGCTGGGATACTTTTGT	CTCGGCTGCTGCATTGTTC
h-BCL2	CATGTGTGTGGAGAGCGTCAA	GCCGGTTCAGGTACTCAGTCA
h-CYP2C8	CACCCAGAGGTCACAGCTAAAGT	CATGTGGCTATCCTGCAT
h-CYP2C9	GCCTGAAACCCATAGTGGTG	GGGGCTCAAAATCTTGATG
h-CYP2C19	TGCTCTCCTTCTCCTGCTGAA	TGCCAACGACACGTTCAATC

## Data Availability

All of the data described in the study are available from the corresponding author upon reasonable request. All other authors had full access to the study’s data and took responsibility for its integrity and accuracy.
